# Chemoprevention of Colonic Aberrant Crypt Foci by Novel Schiff Based Dichlorido(4-Methoxy-2-{[2-(Piperazin-4-Ium-1-Yl)Ethyl]Iminomethyl}Phenolate)Cd Complex in Azoxymethane-Induced Colorectal Cancer in Rats

**DOI:** 10.1038/srep12379

**Published:** 2015-07-23

**Authors:** Maryam Hajrezaie, Keivan Shams, Soheil Zorofchian Moghadamtousi, Hamed Karimian, Pouya Hassandarvish, Mozhgan Emtyazjoo, Maryam Zahedifard, Nazia Abdul Majid, Hapipah Mohd Ali, Mahmood Ameen Abdulla

**Affiliations:** 1 Department of Biomedical Science, Faculty of Medicine, University of Malaya, 50603 Kuala Lumpur, Malaysia; 2 Institute of Biological Science, Faculty of Science, University of Malaya, 50603 Kuala Lumpur, Malaysia; 3 Department of Biology, Islamic Azad University North Tehran Branch, 1841914497 Tehran, Iran; 4 Department of Chemistry, University of Malaya, 50603 Kuala Lumpur, Malaysia

## Abstract

Schiff-based complexes as a source of cancer chemotherapeutic compounds have been subjected to the variety of anticancer studies. The *in-vitro* analysis confirmed the CdCl_2_(C_14_H_21_N_3_O_2_) complex possess cytotoxicity and apoptosis induction properties in colon cancer cells, so lead to investigate the inhibitory efficiency of the compound on colonic aberrant crypt foci (ACF). Five groups of adult male rats were used in this study: Vehicle, cancer control, positive control groups and the groups treated with 25 and 50 mg/kg of complex for 10 weeks. The rats in vehicle group were injected subcutaneously with 15 mg/kg of sterile normal saline once a week for 2 weeks and orally administered with 5% Tween-20 (5 ml/kg) for 10 weeks, other groups were injected subcutaneously with 15 mg/kg azoxymethane once a week for 2 weeks. The rats in positive groups were injected intra-peritoneally with 35 mg/kg 5-Flourouracil four times in a month. Administration of the complex suppressed total colonic ACF formation up to 73.4% (*P* < 0.05). The results also showed that treatment with the complex significantly reduced the level of malondialdehyde while increasing superoxide dismutase and catalase activities. Furthermore, the down-regulation of PCNA and Bcl2 and the up-regulation of Bax was confirmed by immunohistochemical staining.

Although there have been noteworthy achievements in decreasing cancer incidence recently, a sizeable proportion of the world population are still afflicted with cancers^1^. In the United States, one in two men and one in three women will experience cancer during their lifespan.

Despite considerable progress in clarifying the critical factors involved in the prevention of different types of cancers, colorectal cancer it is still the third and second most prevalent cancer among women and men in the United States respectively^2^.

Dietary changes, including intake of vegetables, fresh fruits and plants with high rates of natural antioxidants could prevent 70–80% of colorectal cancer^3^. Furthermore, obesity also increases the risk for colorectal cancer, while physical activities have reverse effects^4^. More than 50% of patients suffering from colon cancer have locally advanced disease at the time of prognosis and in spite of surgical treatment and adjuvant chemotherapy, approximately one third of patients will develop recurrence of the disease^5^. Additionally, most of the patients treated with available therapeutic treatments, including radiotherapy, chemotherapy and surgery, are afflicted with severe side effects, namely hair loss, immunosuppression, diarrhoea and bleeding^6^. Thus, there is an urgent interest and demand in using inorganic chemical substance to identify novel chemotherapeutic agents that are more effective and have minimal adverse side effects.

To successfully implement cancer control in individuals at elevated risk for cancer, a mechanism is needed to rapidly evaluate the potential agents for further studies in clinical trials. Induction of tumorigenesis through the administration of the carcinogen of azoxymethane (AOM) in rat colons is a well-utilised system for efficacy testing in colon cancer researches^7^. AOM or methyl-methylimino-oxidoazanium is the oxide form of azoxymethane with a chemical formula of C_2_H_6_N_2_O which induces colon cancer with high similarity in comparison to the pathogenesis of human sporadic colon cancer^8^,^9^. AOM induces early lesions in the left colon, which normally involves in lymphoid aggregates^10^,^11^. All known colon carcinogens induce aberrant crypt foci (ACF) which is a preneoplastic lesions in rat colons, that induces molecular mutations in regulatory genes in the development of colon cancer^12^. ACF characterisation capitalises on the multistage development of colon cancer which is common to humans and rats^13^. In order to provide economical and simple tool for preliminary investigation of potential chemopreventive agents, the use of the ACF system for colon cancer studies has recently accelerated. Concurrently, other premalignant lesions have also been identified in the rat colon as possible biomarkers for further studies^14^.

Schiff bases are a critical group of compounds because they are being utilised as starters in the synthesis of industrial products and chelating ligands in the field of organic chemistry^15^,^16^. Schiff base compounds with O, S and N donors have formation similarities with neutral biological systems and are utilised to examine the conversion mechanisms of racemisation reactions in the biological systems due to the presence of an imine group^17^. Furthermore, they show a variety of bioactivity functions including anti-ulcer^18^, antibacterial and antifungal^19^,^20^, anti-diabetic^21^, antitumor^22^,^23^, anti-proliferative^24^ and anti-inflammatory activities^25^.

The present study was designed to observe the inhibitory effects of the CdCl_2_(C_14_H_21_N_3_O_2_) complex on AOM-induced colon carcinogenesis in rats focusing primarily in ACF incidences.

## Results

### Acute toxicity study

**Figure 1:**
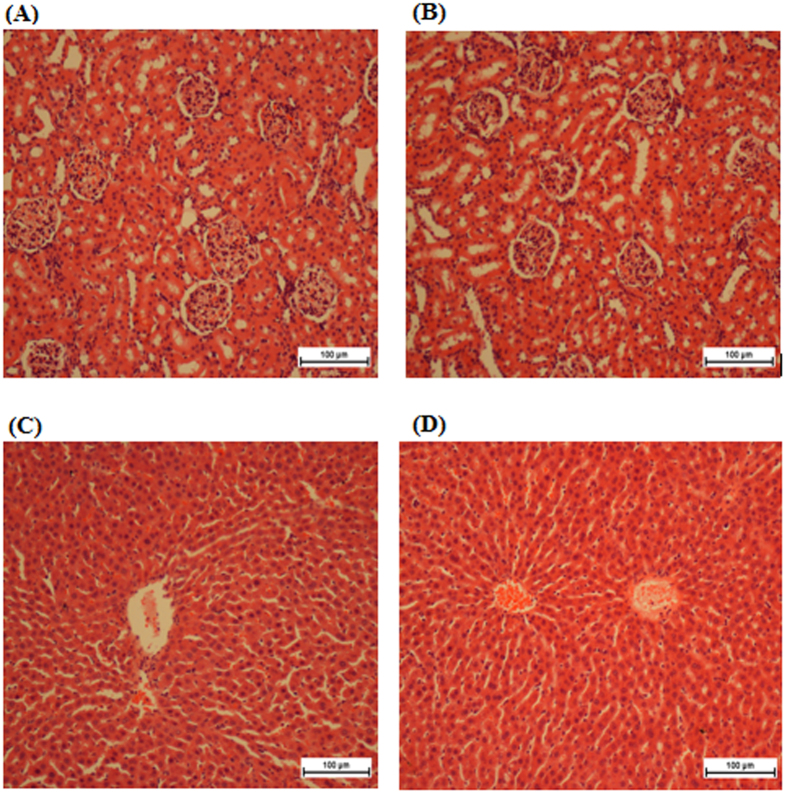
Histological sections of liver (first row) and kidney (second row) in female rats. (**A**,**D**) Control pre-treated with vehicle (10% Tween-20), (**B**,**E**) pre-treated with 250 mg/kg of CdCl_2_(C_14_H_21_N_3_O_2_) complex in female group. No structural differences were observed between the compound treated group and the control group. (**H,E**) stain; 100 × magnification.

**Figure 2:**
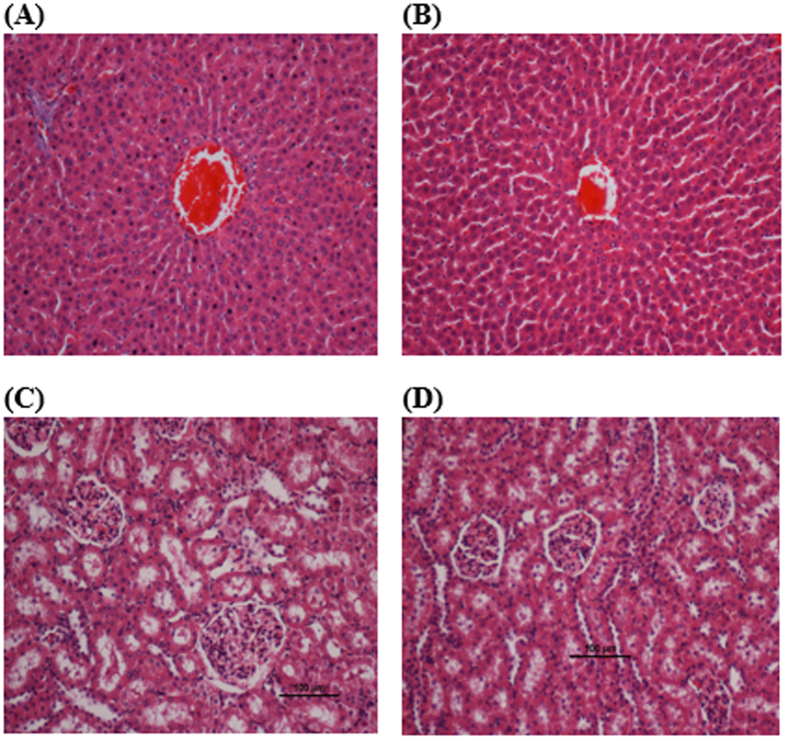
Histological sections of liver (first row) and kidney (second row) in male rats. (**A**,**D**) Control pre-treated with vehicle (10% Tween-20), (**B**,**E**) pre-treated with 250 mg/kg of CdCl_2_(C_14_H_21_N_3_O_2_) complex in male group. No structural differences were observed between the compound treated group and the control group. (**H**,**E**) stain; 100 × magnification.

Acute toxicity results revealed that there was no significant toxicity effect when a dosage of 250 mg/kg of the compound was administered to the rats. There were no differences in terms of behaviour, clinical observations and mortality (Table S1), body weight (Table S2), renal and liver functions (Tables 1 and 2) and histopathology evaluation in female (Fig. 1) and male rats (Fig. 2).

### Rat’s body weight and serum biochemistry analysis of the chemopreventive potential of the CdCl_2_(C_14_H_21_N_3_O_2_)

There were no significant differences in rat body weight in any of the groups when compared with their respective vehicle groups (Table 3). Parameters for liver and renal function for all groups were analysed and compared with the vehicle groups (Tables 4 and 5).

### Counting the ACF

**Figure 3:**
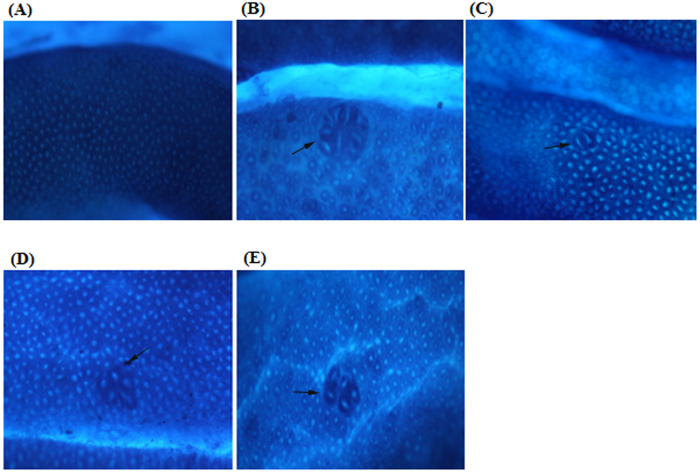
Topographic views of colon mucosa. (**A**) Vehicle colon mucosa, (**B**) cancer control group, (**C**) positive control group, (**D**) treatment group with 25 mg/kg CdCl_2_(C_14_H_21_N_3_O_2_) complex, (**E**) treatment group with 50 mg/kg CdCl_2_(C_14_H_21_N_3_O_2_) complex in methylene blue staining of rat colonic tissue. ACF were distinguished from the surrounding of normal crypts by their increased in size, increased distance from lamina to basal surfaces of cells and easily discernible pericryptalzone. Magnification, ×20.

Following staining with methylene blue, no ACF were observed in the control groups, whereas all animals in the cancer control group developed ACF. The CdCl_2_(C_14_H_21_N_3_O_2_) complex induced inhibition up to a 73.4% (*p* < 0.05) in treated rats compared with the cancer control group. The average total number of ACF as well as the number of crypts per focus were obtained (Table 6). Figure 3 illustrates the topographic views of normal colon mucosa in the rat colonic tissues stained in methylene blue within different groups. ACF were mostly shown to be dispersed in the central of the rats colon when injected with AOM alone (Table 7). The Schiff base compounds significantly decrease the number of ACF in the distal, middle and proximal of the colon in comparison with the AOM treated group (*p* < 0.05).

### Histological analysis of aberrant crypt foci

**Figure 4:**
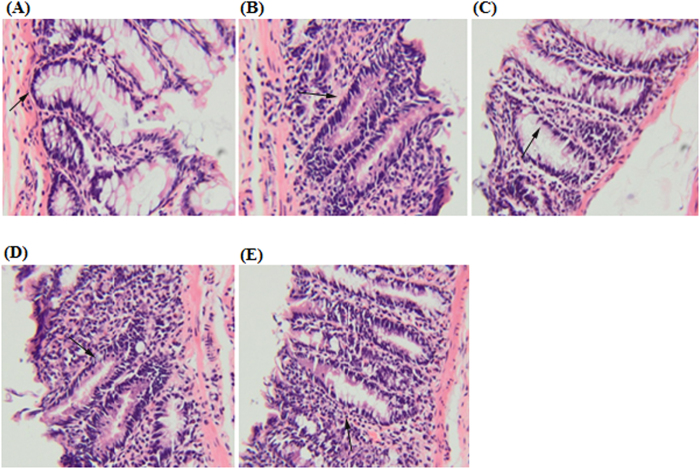
Histological study of colon cancer in rats. (**A**) Vehicle colon mucosa, (**B**) cancer control group, (**C**) positive control group, (**D**) treatment group with 25 mg/kg CdCl_2_(C_14_H_21_N_3_O_2_) complex, (**E**) treatment group with 50 mg/kg CdCl_2_(C_14_H_21_N_3_O_2_) complex. The section was cut parallel to the muscle layer. (**H**,**E**)stain; 100 × magnification).

Based on the images of haematoxylin and eosin staining of dysplastic ACF and normal colon cells, elongated nuclei, loss of cell polarity, increased mitotic activity, lack of goblet cells and narrow lumens in epithelial cells of ACF were observed when compared with the surrounding normal crypts. The normal control group showed circular shaped cells and basal locations of the nuclei. Histological images of the colon tissues from the positive control group showed decrease in the numbers of cells with pathological changes and similar inhibition was observed in CdCl_2_(C_14_H_21_N_3_O_2_) complex treatment groups (Fig. 4).

### Immunohistochemistry

**Figure 5:**
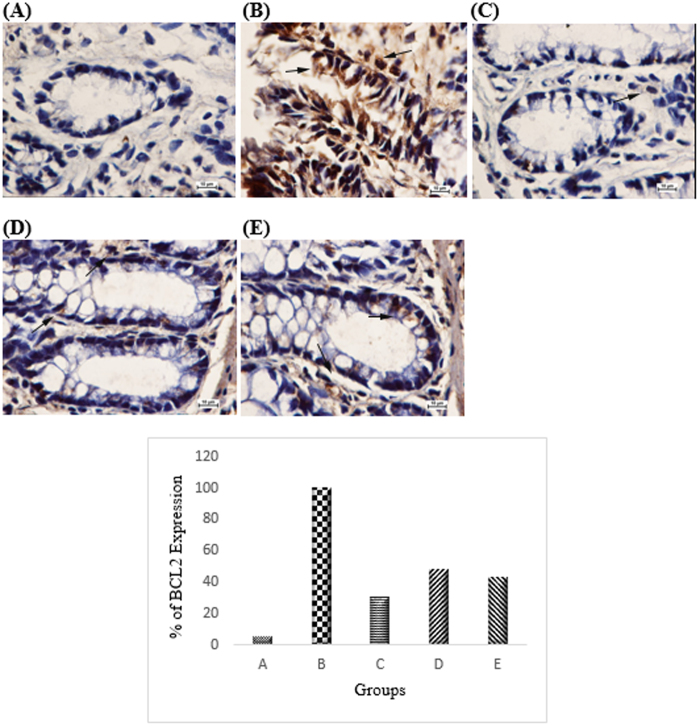
Immunohistochemical analysis of expression of Bcl2 in the colon of different group of rats. (**A**) Normal colon mucosa. (**B**) Colon mucosa of the group exposed to AOM. (**C**) Colon mucosa of the group treated with 5-fluorouracil., (**D**) Colon mucosa of the treatment group with 25 mg/kg CdCl_2_(C_14_H_21_N_3_O_2_) complex, (**E**) Colon mucosa of the treatment group with 50 mg/kg CdCl_2_(C_14_H_21_N_3_O_2_) complex. Immunohistochemistry staining showed upregulation of Bcl2 protein expression in rats treated with CdCl_2_(C_14_H_21_N_3_O_2_) Schiff base compounds. Magnification, ×100.

**Figure 6:**
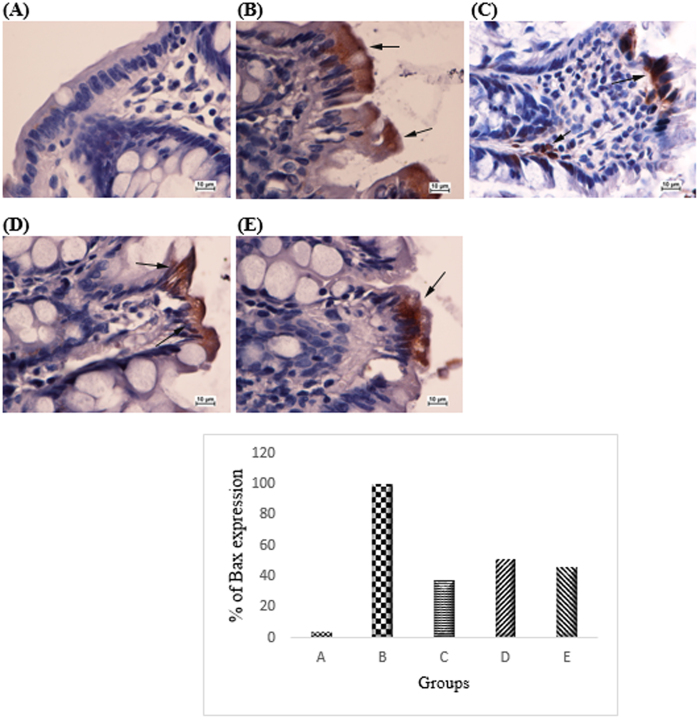
Immunohistochemical analysis of expression of Bax in the colon of different group of rats. (**A**) Normal colon mucosa. (**B**) Colon mucosa of the group exposed to AOM. (**C**) Colon mucosa of the group treated with 5-fluorouracil. (**D**) Colon mucosa of the treatment group with 25 mg/kg CdCl_2_(C_14_H_21_N_3_O_2_) complex, (**E**) Colon mucosa of the treatment group with 50 mg/kg CdCl_2_(C_14_H_21_N_3_O_2_) complex. Immunohistochemistry staining of Bax proteins showed downregulation of expression of Bax protein in rats treated with CdCl_2_(C_14_H_21_N_3_O_2_) Schiff base compounds. Magnification, ×100.

**Figure 7:**
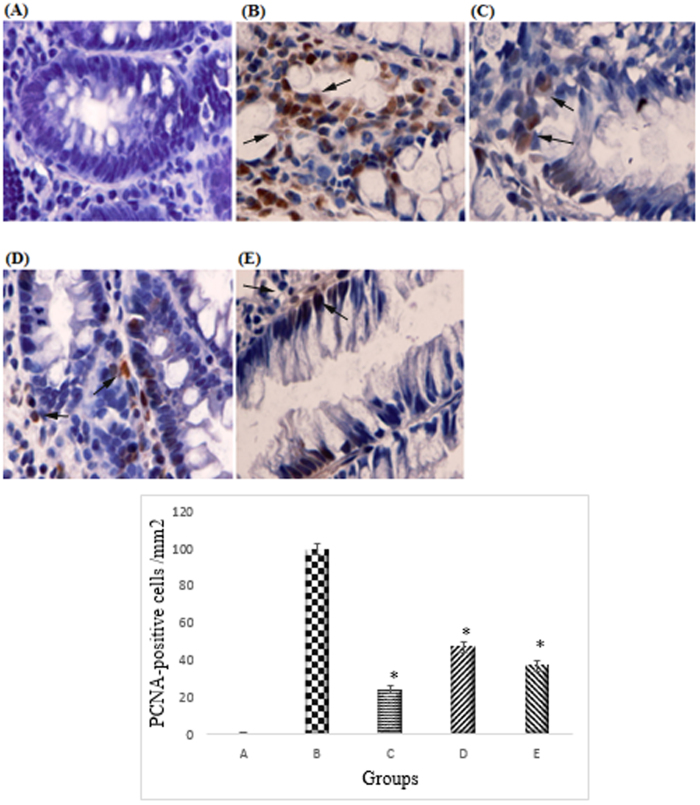
Immunohistochemical analysis of expression of PCNA in the colon of different group of rats. (**A**) Normal colon mucosa. (**B**) Colon mucosa of the group exposed to AOM. (**C**) Colon mucosa of the group treated with 5-fluorouracil. (**D**) Colon mucosa of the treatment group with 25 mg/kg CdCl_2_(C_14_H_21_N_3_O_2_) complex, (**E**) Colon mucosa of the treatment group with 50 mg/kg CdCl_2_(C_14_H_21_N_3_O_2_) complex. Immunohistochemistry staining of PCNA proteins showed downregulation of PCNA protein in rats treated with CdCl_2_(C_14_H_21_N_3_O_2_) Schiff base compounds. Magnification, ×100. All values were expressed at ± standard error of mean. The mean differences were significant at p < 0.05 when compared to the cancer control group.

Based on the immunohistochemistry analysis Bcl2 was up-regulated in the cancer control group (Fig. 5). The CdCl_2_(C_14_H_21_N_3_O_2_) complex treatment group caused down-regulation of anti-apoptotic protein Bcl-2 by inducing anti-proliferative effects in the cancer control groups (47% and 43% respectively in groups treated with 25 mg/kg and 50 mg/kg of complex). Pro-apoptotic protein Bax was up-regulated in the AOM-induced group treated with the complex compare to the AOM control group which was down-regulated (51% and 46% respectively in groups treated with 25 mg/kg and 50 mg/kg of complex) (Fig. 6). PCNA was evaluated as a marker for cell proliferation in the colon specimens. Immunohistochemical of PCNA staining of the colon sections from the cancer group revealed higher number of positive cells than those from the AOM treated groups. The PCNA-positive cells (%) of the colon tissue in the cancer control group were 100%, whereas PCNA-positive cells (%) from the treated group were 47.4% and 35.4%, respectively (*p* < 0.05) (Fig. 7).

### Antioxidant activities of homogenised colon

**Figure 8:**
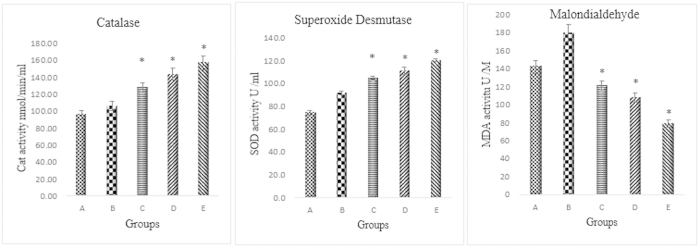
Effects of the compound on antioxidant enzyme activities in homogenised tissue. (**A**) Vehicle colon group, (**B**) group exposed to AOM, (**C**) group treated with 5-fluorouracil, (**D**) treatment group with 25 mg/kg CdCl_2_(C_14_H_21_N_3_O_2_) complex, (**E**) treatment group with 50 mg/kg CdCl_2_(C_14_H_21_N_3_O_2_) complex. All values were expressed at ± standard error of means. Mean differences were significant at p < 0.05 when compared with cancer control group.

The results of this study demonstrated that the levels of SOD and CAT in the homogenised colon treated with the CdCl_2_(C_14_H_21_N_3_O_2_) complex significantly (*p* < 0.05) increased when compared with the cancer control group. The MDA level of the homogenised colon tissue was significantly (*p* < 0.05) decreased relative to the cancer control group (Fig. 8).

## Discussion

New anticancer drugs based on metal complexes and with an efficient administration that focuses on the induction of apoptosis in cancer cells provides significant improvements for pharmacological studies^26^. This development was significantly driven by platinum-based antitumor agents such as oxaliplatin, cisplatin and carboplatin; however, severe adverse side effects were associated with the use of these drugs. As mentioned in few studies, Schiff based complexes are a well-known groups where numerous metal derivative of these group are reported for their bioactivities against microbes^27^, fungi^28^ and cancers^29^. Their active metal groups and ligands are mainly attributed to their therapeutic activities^30^,^31^. Based on the promising anticancer role of CdCl2(C14H21N3O2) complex on HT-29 cells which was supported through different mechanistic pathways such as release of lactate dehydrogenase (LDH), reactive oxygen species (ROS) production, effect on mitochondria membrane potential (MMP), caspase activation which was conducted by our group^32^, we planned to proceed on *in-vivo* analysis. It is critical to report the safety and the proper and efficient route for the administration of these novel anticancer agents for the development of drugs based on the global harmonised system of classification and labelling of chemicals^33^,^34^. The results of the acute toxicity tests demonstrated no abnormalities in behaviour, loss of body weight and renal/liver function differences between the treated groups and the normal control group, thereby confirming the safe usage of the CdCl_2_(C_14_H_21_N_3_O_2_) complex for chemopreventive effects against colon cancer. The total amount of the compound was too little and because we found no toxicity effect from the acute toxicity evaluation based on 250 mg/kg, we chose one-tenth of that selected dosage and the other high dose was twice that of one-tenth dose.

As shown from the results, AOM caused colon cancer confirmed by the appearances of aberrant crypt foci, an early stage of biomarker^35^,^36^,^37^,^38^. ACF induced by AOM in rodents was reported by Bird in 1987 and in humans by Pretlow in 1991^39^,^40^. ACF induced by AOM has been widely used in animal studies^23^,^41^. The results of the ACF scoring analysis proved that the CdCl_2_(C_14_H_21_N_3_O_2_) complex significantly inhibited ACF formation in male rats. Haematoxylin and eosin staining of ACF illustrated the cell deformation from atypia to dysplasia^42^. When compared with the cancer control group, the histology evaluation of colon tissue also clearly showed the prevention of dysplasia and colon damage and the reduction in induced inflammation and tissue necrosis in the treated group, similar to the results of groups treated with 5-fluorouracil. According to Hajrezaie *et al.*^23^ research funding the same protective efficiency was observed from the Schiff based compounds.

AOM causes an imbalance in the level of ROS in colon cells leading to oxidative injuries. The perturbation in the level of ROS has significant effects on apoptosis pathways and cellular functions^43^,^44^,^45^. As a result of the elevation in the level of superoxide anions and superoxide dismutase; the essential scavenging enzyme, normally converts superoxide anion into hydrogen peroxide, which is then changed to water by either glutathione peroxidase or catalase. We found that the treated groups had significantly higher enzymatic antioxidant defence activity of SOD and CAT when compared with the cancer control group. The mechanism of action of this compound might be through free radical scavenging and quenching of the formation of singlet oxygen, which protects the colon against oxidative stress and stimulates colon repair mechanisms. There is a possibility that the compound possesses protective effects against ACF formation and colon injuries through the endogenous oxidative enzyme systems which is involved in the colon defense system, such as CAT and GPX, which counterbalance the oxidative stress induced by AOM. Previous studies also showed increased activities of antioxidant enzymes, which were induced by antitumor agents in chemopreventive therapy^46^. DNA and cellular membrane damages caused by increased levels of ROS as a result of colon cancer progression leads to elevated levels of lipid peroxidation. Malondialdehyde (MDA) is a biomarker for measuring the oxidative stress and is used to determine the level of lipid peroxidation. The results of this study were similar to the results of previous investigations, which revealed increased levels of MDA in cancer control rats. The reduction in the MDA level was comparable with the drug treated group^47^,^48^,^49^,^50^.

Uncontrolled cell division is indicative of the initial stage of cancer disease. PCNA protein is an indicator for presence of different cancers. Studies in animal models showed that abnormal epithelial cell proliferations are one of the earliest indications of pre-neoplasia^51^,^52^. Based on a study by Deschner EE *et al.*^53^, animals treated with any chemical colon carcinogen revealed a larger proliferation zone and a higher labeling index when compared with the vehicle-treated group. Our results revealed that the CdCl_2_(C_14_H_21_N_3_O_2_) complex exhibited inhibitory effects against ACF productions via the suppression of PCNA expression when compared with the cancer control group. The same reduction in the expression of PCNA was previously reported by chemopreventive investigations against AOM-induced colon cancer in animal models^54^,^55^,^56^,^57^. Regulation of cell proliferation by CdCl_2_(C_14_H_21_N_3_O_2_) complex activities can be considered as one of the possible mechanisms behind the inhibitory effect against ACF reductions and colon cancer prevention. Therefore, the groups treated with the compound had a smaller proliferation zone and a lower labeling index, as the cells were no longer in the growth cycle (*p* < 0.05).

The expression analyses of Bax (BCL2-associated X protein) and Bcl-2 (B-cell lymphoma protein 2) were investigated in different groups. Each protein has an important role in the cancer prevention mechanism and it is essential for normal cells to have balanced level of expression of these proteins. Cell death is controlled via the inhibition of various apoptotic signals by Bcl-2, while Bax releases apoptosis-promoting factors to the cytoplasm^58^. The results of our study revealed that the CdCl_2_(C_14_H_21_N_3_O_2_) complex increased the expression of BAX protein, while the expression of Bcl-2 protein was down-regulated. The previous studies on chemoprevention against colon cancer also revealed the same pattern of Bax and Bcl-2 protein expression^59^,^60^.

## Conclusions

In conclusion, the CdCl_2_(C_14_H_21_N_3_O_2_) complex revealed the nontoxic oral administration and chemopreventive effect against AOM-induced colon cancer rats via the suppression of ACF formation in the distal, middle and proximal sections of the colon. Reduction in damage induced by AOM in treated rats was confirmed by the microscopic evidences which demonstrated significant chemopreventive activity in colon tissues. The mechanisms of this chemoprevention included the down-regulation of cell proliferation-promoting proteins in cancer cells (which was demonstrated by PCNA immunohistochemistry) and the elevation of the levels of antioxidant enzymes which protect colon cells from oxidative injuries caused by the injection of azoxymethane. The complexes also activates apoptosis via the mitochondrial pathway by down-regulating Bcl-2 and up-regulating of Bax.

## Materials and methods

### Chemicals

In this experiment, the carcinogenic chemical AOM was purchased from Sigma Aldrich (St. Louis, MO, USA). 5-fluorouracil, a colon cancer drug reference, was purchased from Calbiochem (San Diego, CA, USA).

### Preparation of the CdCl_2_(C_14_H_21_N_3_O_2_) complex

The Schiff base compounds were provided from the Chemistry Department, Faculty of Science, University of Malaya and synthesised according to previous procedure reported by Saleh Salga *et al.*^61^.

### Ethical issues

The protocol of this study was approved by the ethics committee for animal experimentation in accordance with Ethic No. PM/28/09/2011/MAA (R). Rats (180–200 g) were provided from the Animal House, Faculty of Medicine, University of Malaya. The animals were maintained and housed separately on a standard libitum, pellet diet and tap water. Human care of animals was based on the criteria noted in the “Guide for the care and use of laboratory animals” prepared by National Academy of Sciences^33^,^34^.

### Acute toxicity evaluation

Acute toxicity was evaluated to determine the safe dosage of the CdCl_2_(C_14_H_21_N_3_O_2_) complex. 24 healthy female and male mice were sorted into 2 4 groups: a vehicle control group and a the two groups treated with 250 mg/kg of the compound^62^. Prior oral administration of the compound, the mice were fasted overnight. To detect clinical and toxicological signals after feeding, the animals were observed for the first 30 min and followed by 2, 4, 24 and 48 h. Food was given 3 to 4 h after the respective dosage was administered. The animal’s weight were recorded during the experimental time. The mice were euthanized with an over dose of xylazine and ketamine anaesthesia. Animals were all sacrificed after 15 days. Bloods were collected in gel-activated clot tubes which were then sent for serum biochemical analysis. Haematoxylin and eosin (H&E) staining for histological evaluations of the kidney and liver were also conducted.

### Experimental procedure for evaluation of the chemopreventive effect of CdCl_2_(C_14_H_21_N_3_O_2_) complex

Thirty adult male Sprague Dawley rats were divided randomly into the following 5 groups of 6 rats each: (group 1) vehicle control group (these rats received subcutaneous injections of normal saline once a week for 2 weeks and were orally administered 10% Tween 20 each day for 10 weeks), (group 2) cancer control group (these rats received subcutaneous injections of 15 mg/kg AOM once per week for two consecutive weeks), (group 3) positive control group (these rats received subcutaneous injections of 15 mg/kg AOM once per week for two consecutive weeks and intra-peritoneal injections of 35 mg/kg 5-fluorouracil for 4 weeks) and two treatment groups [these rats received subcutaneous injections of 15 mg/kg AOM once per week for two consecutive weeks and oral administration of CdCl_2_(C_14_H_21_N_3_O_2_) complex at dosages of 25 mg/kg (group 4) and 50 mg/kg (group 5) for 10 weeks]. During these experiments, the rats were weighed and observed daily for signs of toxicity.

After 10 weeks of experiments, the rats were sacrificed as mentioned above and bloods were collected for biochemical analysis to determine functions of enzymes of the liver and kidney. Kidney and liver specimens were fixed in 10% buffered formalin and embedded in paraffin by automated tissue processing machine (Leica, Nussloch, Germany). Kidney and liver sections (5 μm) were stained with haematoxylin and eosin (H&E) for histological evaluation.

### ACF scoring and histological evaluation of the colon lesions

To evaluate the initiation of colon cancer 10 weeks after the injection of AOM, the rats were euthanized with a high dosage of xylazine and ketamine anesthesia. The colons were then removed and flushed with cold phosphate-buffered saline (PBS) and dissected longitudinally from anus to cecum. Colons were stained with 0.2% methylene blue dissolved in saline and observed through a Nikon dissecting microscope with a fibre optic light source. The number of ACF and the crypt multiplicity were determined. The number of crypts in each focus and the location data were recorded at the time of necropsy. 1 cm of colon sections were fixed with formalin and embedded in paraffin and 5 μm sections were stained with haematoxylin and eosin for histological classification.

### Immunohistochemical evaluation

Colon biopsies were fixed in buffered formalin and were processed by a tissue processing automated machine. The sections were deparaffinised, hydrated and subjected to antigen retrieval by immersion in 10 mM citrate buffer (pH 6.0) at 92–95 °C for 20 min; the slides were cooled to room temperature and were subsequently washed in phosphate-buffered saline (PBS). The endogenous peroxidases were blocked with 1% H_2_O_2_ in PBS for 5 min. The parts were rinsed with PBS and blocked with 3% regular horse serum. The slides were rinsed and incubated with Bcl2 (1: 500, Cat: ab18210), Bax (1: 200, Cat: ab5714) and diluted mouse proliferating cell nuclear antigen (PCNA) (1: 200, Cat: ab2426) monoclonal antibody. The slides were then rinsed with PBS and were incubated with biotinylated secondary anti-mouse immunoglobulin G (IgG) (1: 200 dilution) at 37 °C for 30 min using the ABC staining system. Five sections of the colons of each rat were stained and 1,500 cells were counted per section. The PCNA labelling index (PI) was computed as the [(number of positive cells)/(total number of epithelial cells)] × 100 for each field. These PI values for all different colon sections of the rats belonging to the same group were averaged.

### Antioxidant activity of homogenised colon tissue

After washing the colon tissue samples with ice-cold saline, a homogenate (10% w/v) was prepared in ice-cold 50 mM phosphate buffer (pH 7.4) containing mammalian protease inhibitor cocktail and was centrifuged at 10,000 × g for 30 minutes at 4 °C. The supernatant was used to analyse the activity of catalase (CAT) and superoxide dismutase (SOD) enzymes and for malondialdehyde (MDA) assay. The SOD assay was performed by using the kit from Cayman Chemical Company (Item No. 703202). According to the manufacturer’s protocol, all the reagents and standard dilution were prepared and 10 μl of the samples and standard were pipetted into the 96 wells plate followed by adding the SOD reagent. The plate was led for incubation for 20 minutes at room temperature with gentle shake. The plate was read at 440–460 nm and the SOD value was calculated based on the formula suggested in the manufacturer’s protocol. The SOD assay was performed in the duplicate for the samples and standards where the final volume of the each well was 230 μl. The CAT assay was also performed based on the Cayman Chemical Company kit (Item No. 703202). Briefly, all the reagents and standard were prepared as mentioned according to the manufacturer’s protocol where 20 μl of samples and standard were pipetted in duplicates into the 96 wells plated. All the reagents were added accordingly into the plate followed by incubation as mentioned in the protocol. The absorbance of the samples of the 96 well plate were read at 540 nm wavelength. The Catalase assay was performed at a final volume of 240 μl in all the wells and the Catalase value of each samples were calculated according manufacturer’s protocol. In order to evaluate the level of lipid peroxidation in the mucus membrane, the level of malondialdehyde was estimated using the thiobarbituric acid-reactive substances (as indicators of lipid peroxidation). Briefly the colon tissue homogenates were mixed with solution (0.125 ml) containing 26 mM thiobarbituric acid, 0.26 M HCl, 15% trichloric acid and 0.02% butylatedhydroxytoluene. The mixtures were heated at 96 °C for 15 min and centrifuged at 4,000 rpm for 10 min. The supernatant was transferred to a 96-well plate and the absorption was measured at 532 nm using tetramethoxypropane as standard in the spectrophotometer.

### Statistical analysis

Predictive Analysis Software (PASW) version 18 was used in analysing the data obtained from the study. The experimental data were analysed using variance analysis (one-way ANOVA) plus Tukey’s post-hoc test. Data were calculated and shown as the means ± (standard error of the mean) S.E.M. The level of significance was set as *P* < 0.05.

## Additional Information

**How to cite this article**: Hajrezaie, M. *et al.* Chemoprevention of Colonic Aberrant Crypt Foci by Novel Schiff Based Dichlorido(4-Methoxy-2-{[2-(Piperazin-4-Ium-1-Yl)Ethyl]Iminomethyl}Phenolate)Cd Complex in Azoxymethane-Induced Colorectal Cancer in Rats. *Sci. Rep.* **5**, 12379; doi: 10.1038/srep12379 (2015).

## Supplementary Material

Supplementary Information

## Figures and Tables

**Table 1:** Effects of 250 mg/kg of CdCl_2_(C _14_H_21_N_3_O_2_) complex on renal function test.

Groups	Sodium (mmo/L)	Pottasium (mmol/L)	Chloride (mmol/L)	CO_2_(mmol/L)	Anion gap (mmol/L)	Urea (mmol/L)	Creatinine (μmol/L)
Vehicle Male	146 ± 1.1	4.8 ± 0.06	105.0 ± 0.3	26.9 ± 34	17.4 ± 0.5	5.7 ± 0.3	34.95 ± 3.4
CdCl_2_(C_14_H_21_N_3_O_2_) complex (250 mg/kg)	140 ± 1.58	4.7 ± 0.1	105.1 ± 0.45	26.28 ± 0.53	18.4 ± 0.7	5.8 ± 0.11	35.6 ± 3.4
Vehicle Female	144.6 ± 1.2	4.6 ± 0.12	105 ± 0.27	26.3 ± 0.24	18.0 ± 0.7	5.5 ± 0.2	35.2 ± 2.7
CdCl_2_(C_14_H_21_N_3_O_2_) complex (250 mg/kg)	143 ± 0.55	4.8 ± 0.1	105.2 ± 0.63	25.3 ± 0.85	18.0 ± 0.8	5.2 ± 0.7	35.3 ± 1.7

Values are expressed as means ± S.E.M. There are no statistically significant differences between the measurements among different groups. Significance was set at *P* < 0.05.

**Table 2:** Effects of 250 mg/kg of CdCl_2_(C_14_H_21_N_3_O_2_) complex on Liver function test.

Groups	Total protein (g/L)	Albumin (g/L)	Globulin (g/L)	TB (μmol/L)	CB (μmol/L)	AP (IU/L)	ALT (IU/L)	AST (IU/L)	GGT (IU/L)
Vehicle Male	70.3 ± 3.8	10.5 ± 0.51	60.2 ± 2.5	3.0 ± 0.0	1.0 ± 0.0	98.7 ± 5.2	70.4 ± 3.8	227 ± 11.7	4.0 ± 0.27
CdCl_2_(C_14_H_21_N_3_O_2_) complex (250 mg/kg)	71.5 ± 4.1	10.7 ± 0.93	60.0 ± 0.83	3.0 ± 0.0	1.0 ± 0.0	91.4 ± 4.8	69.8 ± 2.2	227.4 ± 11.4	3.9 ± 0.09
Vehicle Female	72.0 ± 2.1	10.6 ± 0.83	59.8 ± 1.7	3.0 ± 0.0	1.0 ± 0.0	99.6 ± 6.1	65.3 ± 4.1	232 ± 12.4	3.9 ± 0.13
CdCl_2_(C_14_H_21_N_3_O_2_) complex (250 mg/kg)	69.5 ± 1.95	10.4 ± 0.88	58.2 ± 2.6	3.0 ± 0.0	1.0 ± 0.0	102.7 ± 3.6	69.8 ± 5.1	235.7 ± 11.4	3.7 ± 0.03

Values are expressed as means ± S.E.M. There are no statistically significant differences between the measurements of different groups. Significance was set at *P* < 0.05.

**Table 3:** Effects of CdCl_2_(C_14_H _21_N_3_O_2_) complex on the rat’s body weight in AOM-induced colon cancer study.

Group	week 1	week 10
Vehicle control group	148 ± 6.4	363.3 ± 11.5
Cancer control group	152.4 ± 4.9	372.2 ± 16.8
Positive control group	151 ± 6.3	365 ± 13.8
Low dosage of complex (25 mg/kg)	156.6 ± 5.2	383.4 ± 18.3
High dosage of complex (50 mg/kg)	160.2 ± 7.7	380.6 ± 14.9

Values are expressed as the means ± S.E.M. There are no statistically significant differences between the measurements of different groups. Significance was set at P < 0.05.

**Table 4:** Effects of CdCl_2_(C_14_H_21_N_3_O_2_) complex on liver function parameter in AOM-induced colon cancer study.

Group	Total protein (g/L)	Albumin (g/L)	AST (IU/L)	ALT (IU/L)	GGT (IU/L)
Vehicle control group	66.32 ± 0.44	11.3 ± 0.89	206.2 ± 2.48	61 ± 2.33	5.4 ± 0.3
Cancer control group	69.5 ± 39	10.27 ± 0.54	210.0 ± 3.60	66.2 ± 1.5	3.7 ± 0.6
Positive control group	73.6 ± 2.1	11.3 ± 0.89	182.0 ± 4.5	51.0 ± 0.87	6.1 ± 0.48
Low dosage of complex (25 mg/kg)	68.5 ± 0.67	11.4 ± 0.3	230.5 ± 6.1	70.5 ± 0.63	3.0 ± 0.1
High dosage of complex (50 mg/kg)	70.9 ± 1.1	11.0 ± 0.52	221 ± 3.8	61.5 ± 1.8	3.0 ± 0.1

Values are expressed as the means ± SEM There are no significant differences between groups. Significance was set at P < 0.05 compared with cancer control group. TB: total bilirubin; ALT: alanine aminotransferase; AST: aspartate aminotransferase; GGT: G-glutamyl transferase.

**Table 5:** Effects of CdCl2(C14H21N3O2) complex on renal function parameters in AOM-induced colon cancer study.

Group	Urea (mmol/L)	Creatinine (μmol/L)
Vehicle control group	5.44 ± 0.20	53.4 ± 5.32
Cancer control group	5.6 ± 0.03	33.6 ± 0.47
Positive control group	5.5 ± 0.02	43.2 ± 0.64
Low dosage of complex (25 mg/kg)	5.4 ± 0.2	35.23 ± 1.3
High dosage of complex (50 mg/kg)	5.2 ± 0.5	34.6 ± 1.4

**Table 6:** Effect of CdCl_2_(C_14_H_21_N_3_O_ 2_) complex on AOM-induced colonic ACF containing four or more aberrant crypts in rats.

	No. of crypts per ACF					
Treatment group	1 crypt	2 crypt	3 crypt	4 crypt and more	Total ACF	Inhibition %
Vehicle control group	0.0 ± 0.0	0.0 ± 0.0	0.0 ± 0.0	0.0 ± 0.0	0.0 ± 0.0	0.0 ± 0.0
Cancer control group	9 ± 0.43	27 ± 3.27	21 ± 3.27	30 ± 1.27	88 ± 6.7	0
Positive control group	2 ± 0.03*	6 ± 0.69*	5 ± 0.64*	10 ± 0.26*	22 ± 2.81*	75.2*
Low dosage of complex (25 mg/kg)	2 ± 0.71*	5 ± 0.14*	5 ± 0.19*	14 ± 1.04*	26 ± 2.70*	71.3*
High dosage of complex (50 mg/kg)	2 ± 0.41*	5 ± 0.52*	4 ± 0.27*	13 ± 1.13*	24 ± 1.53*	73.4*

Values are expressed as the means ± S.E.M. Significance was set at P < 0.05, compared with cancer control group.

**Table 7:** Effect of CdCl_2_(C_14_H_21_N_3_O_2 _) complex on regional distribution of colonic ACF in AOM-induced colonic cancer in rats.

	ACF Counting			
Groups	Proximal	Middle	Distal	Total
Vehicle control group	0.0 ± 0.0	0.0 ± 0.0	0.0 ± 0.0	0.0 ± 0.0
Cancer control group	25 ± 3.66	43 ± 5.77	11 ± 3.79	88 ± 6.7
Positive control group	1 ± 0.0*	18 ± 2.37*	2 ± 0.0*	22 ± 2.81*
Low dosage of complex (25 mg/kg)	8 ± 1.04*	15 ± 7.89*	3 ± 0.5*	26 ± 2.70*
High dosage of complex (50 mg/kg)	1 ± 0.02*	20 ± 4.84*	3 ± 0.3*	24 ± 1.53*

Values are expressed as the means ± S.E.M. Significance was set at P < 0.05, compared with cancer control group.
