# Supplementary material for: Chemoprevention of Colonic Aberrant Crypt Foci by Novel Schiff Based Dichlorido(4-Methoxy-2-{[2-(Piperazin-4-Ium-1-Yl)Ethyl]Iminomethyl}Phenolate)Cd Complex in Azoxymethane-Induced Colorectal Cancer in Rats
Source: Sci Rep. 2015 Jul 23;5:12379. doi: 10.1038/srep12379 (PMC4511874; doi:10.1038/srep12379)
Supplement: Supplementary Information [file srep12379-s1.pdf]

# Chemoprevention of Colonic Aberrant Crypt Foci by Novel Schiff Based Dichlorido(4-Methoxy-2-{[2-(Piperazin-4-Ium-1-Yl)Ethyl]Iminomethyl}Phenolate)Cd Complex in Azoxymethane-Induced Colorectal Cancer in Rats

Maryam Hajrezaie[1,2], Keivan Shams[1], Soheil Zorofchian Moghadamtousi[2], Hamed Karimian[1], Pouya Hassandarvish[1], Mozhgan Emtyazjoo[3], Maryam Zahedifard[1,2], Nazia Abdul Majid[2], Hapipah Mohd Ali [4], Mahmood Ameen Abdulla[1]*

**Supplementary Table S1. Effects of 250 mg/kg of $CdCl_2(C_{14}H_{21}N_3O_2)$ complex on mice mortality.** There was no mortality observed in any group of mice.

| Occurrence of mortality | 10 min | 30 min | 3 h | 24 h | 48 h |
|---|---|---|---|---|---|
| **Vehicle Male** | 0/6 | 0/6 | 0/6 | 0/6 | 0/6 |
| **$CdCl_2(C_{14}H_{21}N_3O_2)$ complex (250 mg/kg)** | 0/6 | 0/6 | 0/6 | 0/6 | 0/6 |
| **Vehicle Female** | 0/6 | 0/6 | 0/6 | 0/6 | 0/6 |
| **$CdCl_2(C_{14}H_{21}N_3O_2)$ complex (250 mg/kg)** | 0/6 | 0/6 | 0/6 | 0/6 | 0/6 |

**Supplementary Table S2. Effects of 250 mg/kg of $CdCl_2(C_{14}H_{21}N_3O_2)$ complex on mice body weight.** Values are expressed as the means ± S.E.M. There are no statistically significant differences between the measurements of different groups. Significance was set at $P < 0.05$.

| Dose of compound | Mice body weight (g) Day 0 | Mice body weight (g) Day15 |
|---|---|---|
| **Vehicle Male** | 28.5± 2.66 | 32± 2.83 |
| **$CdCl_2(C_{14}H_{21}N_3O_2)$ complex (250 mg/kg)** | 30.5 ± 2.86 | 34 ± 3.01 |
| **Vehicle Female** | 23 ± 2.37 | 26 ± 2.70 |
| **$CdCl_2(C_{14}H_{21}N_3O_2)$ complex (250 mg/kg)** | 22.5 ± 2.60 | 26 ± 2.77 |
